# Microstructural White Matter Alterations in Cognitively Impaired Patients at Early Stages of Multiple Sclerosis

**DOI:** 10.1007/s00062-021-01010-8

**Published:** 2021-03-31

**Authors:** Ruth Schneider, Britta Matusche, Erhan Genç, Ralf Gold, Barbara Bellenberg, Carsten Lukas

**Affiliations:** 1 Department of Neurology, St. Josef Hospital, Ruhr-University Bochum, Bochum, Germany; 2 Institute of Neuroradiology, St. Josef Hospital, Ruhr-University Bochum, Bochum, Germany; 3 Department of Psychology and Neurosciences, Leibniz Research Center for Working Environment and Human Factors, Dortmund, Germany; 4 Department of Diagnostic and Interventional Radiology and Nuclear Medicine, St. Josef Hospital, Ruhr-University Bochum, Bochum, Germany

**Keywords:** Clinically isolated syndrome, Cognitive impairment, Fractional anisotropy, Mean diffusivity, Tract based spatial statistic

## Abstract

**Purpose:**

As conventional quantitative magnetic resonance imaging (MRI) parameters are weakly associated with cognitive impairment (CI) in early multiple sclerosis (MS), we explored microstructural white matter alterations in early MS or clinically isolated syndrome (CIS) comparing patients with or without CI.

**Methods:**

Based on a preceding tract-based spatial statistics analysis (3 Tesla MRI) which contrasted 106 patients with early MS or CIS and 49 healthy controls, diffusion metrics (fractional anisotropy, FA, mean diffusivity, MD) were extracted from significant clusters using an atlas-based approach. The FA and MD were compared between patients with (Ci_P *n* = 14) and without (Cp_P *n* = 81) cognitive impairment in a subset of patients who underwent CI screening.

**Results:**

The FA was reduced in Ci_P compared to Cp_P in the splenium of corpus callosum (*p* = 0.001), right parahippocampal cingulum (*p* = 0.002) and fornix cres./stria terminalis (0.042), left posterior corona radiata (*p* = 0.012), bilateral cerebral peduncles, medial lemniscus and in cerebellar tracts. Increased MD was detected in the splenium of corpus callosum (*p* = 0.01). The CI-related localizations overlapped only partially with MS lesions.

**Conclusion:**

Microstructural white matter alterations at disease onset were detectable in Ci_P compared to Cp_P in known cognitively relevant fiber tracts, indicating the relevance of early treatment initiation in MS and CIS.

**Supplementary Information:**

The online version of this article (10.1007/s00062-021-01010-8) contains supplementary material, which is available to authorized users.

## Introduction

Cognitive impairment (CI) is a frequent symptom with prevalence of 40–65% in multiple sclerosis (MS). It has been attributed to damage of brain white matter (WM) as well as to brain grey matter (GM), both of which can be caused by demyelination, inflammation and axonal loss [1]. CI has already been reported in early disease stages of MS and clinically isolated syndrome (CIS) with a prevalence ranging from 13% to 20% [2–4]. Due to the fact that CI is highly relevant for occupational disability and restrictions in daily life, the early onset of CI is indicative of future quality of life for MS patients [5]. As a result, efforts have been made to identify risk factors to determine and predict CI based on clinical markers (Expanded disability status sclae (EDSS), disease duration) and conventional magnetic resonance imaging (MRI) parameters (brain lesion load and location in T1 and T2-weighted MRI, brain atrophy) [6, 7]; however, associations between conventional MRI parameters, such as brain atrophy or lesion load, and CI were weak, especially in young MS patients with short disease duration [8]. The lack of association between conventional MRI characteristics and baseline CI was recently confirmed in a large multicenter cohort study on patients first diagnosed with MS or CIS [9].

Thus, there is a need for more sensitive MRI parameters to identify structural brain differences between patients with and without CI, particularly in the early stages of MS. In this context, microstructural tissue damage detectable by diffusion tensor imaging (DTI) seems to be a promising complementary marker in addition to grey matter atrophy [7, 10]. DTI facilitates microstructural WM quantification in normal-appearing WM as well as in MS-related lesions by diffusion metrics, such as fractional anisotropy (FA), mean, axial and radial diffusivity (MD, AD, RD). Both FA and MD are regarded as measures of disrupted fiber integrity and increased mobility of water molecules, while RD and AD have been suggested as markers of myelin and axonal damage, respectively [11, 12].

In order to spatially localize changes of these diffusion-related metrics, tract-based spatial statistics (TBSS) has been developed, which provides voxel-wise statistics on a whole brain skeletonized representation of the major WM fiber tracts [13]. A TBSS study involving long-standing MS demonstrated that patients with CI had more severely altered DTI metrics than cognitively preserved patients. These changes were partly found in regions that are considered specific for CI (i.e., the corpus callosum, CC, cingulum, posterior thalamic tract, superior and inferior longitudinal fasciculus, forceps major, cingulum, fornices) but also in the corticospinal tracts [13]. In patients with CIS or early MS WM tract alterations including corticospinal, interhemispheric and association fiber pathways investigated by TBSS have been reported [14]. In a recent TBSS study of our own group (including 106 patients at first diagnosis of MS or CIS) we detected widespread microstructural alterations compared to healthy controls (HC) in all major white matter pathways including callosal, association, limbic, corona radiata, corticospinal and cerebellar pathways. Herein, similar structures were involved in patients with CIS compared to patients with early MS [15].

On the basis of these previous findings the present study aimed at further characterizing the microstructural differences within these altered regions with respect to their relationship to CI. We therefore classified our patients into cognitively impaired (Ci_P) and cognitively preserved patients (Cp_P) and considered DTI metrics in those regions in which significant WM alterations have been found in our preceding study between patients and HC. We studied differences between patients who were assigned to Ci_P or Cp_P after first diagnosis of MS or CIS to identify relevant clusters in WM which might be related to CI. We hypothesized that even during early disease course of MS or CIS differences in WM integrity between Ci_P and Cp_P will be detected and that in turn microstructural WM damage on fibers related to cognitive processing will be associated to CI.

## Methods

### Patient and Healthy Controls

A total of 106 patients (CIS, *n* = 51) and early relapsing remitting MS (RRMS, *n* = 55) after a first clinical episode of MS were enrolled in a single center taking part in an ongoing prospective longitudinal multicenter cohort study (NationMS) of the German Competence Network Multiple Sclerosis (KKNMS). According to the study design, the enrolled patients were treatment-naive to disease-modifying drugs at study entry and had either a diagnosis of CIS with a high risk of conversion to MS within 6 months, or early definite MS less than 24 months after onset of symptoms [16]. The patients were compared to an age and sex matched healthy control group (HC, *n* = 49). The study was approved by the university local ethics committee (approval No. 3714-10) and all patients provided written informed consent prior to study participation.

A subset of 95 patients who received screening for CI were included in the following analyses of the present study. There were no significant differences regarding age, EDSS or brain lesion load between the groups of patients who did or did not receive cognitive screening. The results are shown in the Online_Resource_1.

### Cognitive Screening and Disability

Screening for CI was performed using the Multiple Sclerosis Inventory for Cognition (MUSIC) [17]. MUSIC is a rapid (about 10–12 min) multiple domain cognitive screening test reflecting the most frequently impaired cognitive domains in MS (verbal fluency, verbal working memory and memory retrieval, visual-spatial processing speed and executive functions), which is widely used in German-speaking countries. A timed verbal fluency task with change of categories reflects verbal fluency and executive functions. Working memory functions and attention are assessed using immediate wordlist recall tasks and memory retrieval using a delayed word list recall tasks. A modified Stroop test (naming of animal silhouettes either in a congruent or incongruent condition) assesses information processing speed and executive functions. For each participant the raw scores are converted into a total sum score (maximum = 30) reflecting general cognitive functioning on the basis of normative data of 158 healthy controls controlling for age, sex and education. The contributions of the subscores to the sum score are weighted on the basis of their respective diagnostic value (superior sensitivity and specificity receiving a higher weight), such that the sum score equally reflects impairment of working memory functions (word recall tasks) and higher executive functions (verbal fluency task and modified Stroop test) [18, 19]. The MUSIC cognition score ranges from 0 to 30, with values ≤ 10 representing marked cognitive dysfunction, 11–15 representing medium grade cognitive dysfunction, 16–20 at least mild cognitive impairment, and > 20 cognitive performance in the normal range. According to the MUSIC rating scale, patients with values higher or equal than 20 were defined as cognitively preserved (Cp_P) while patients with values below 20 were defined as at least mildly cognitive impaired (Ci_P) [20]. Additionally, all patients underwent neurological examinations including the expanded disability status scale (EDSS) [21].

### MR Imaging

Imaging was performed using a single 3T scanner (Achieva Philips, Best, The Netherlands) with a standardized imaging protocol including a single-shot 2D echo planar imaging (EPI) sequence for diffusion-weighted imaging (50 axial slices, 2.5 mm slice thickness, field of view of 320 × 240, in-plane resolution of 2.5 × 2.5 mm^2^, TR/TE 7000/90, flip angle of 90°, 32 gradient directions with b‑value = 900 s/mm^2^ and one volume, two averages, without diffusion weighting, acquisition time of 5min 11s) and an isotropic 3D fluid attenuated inversion recovery (FLAIR) sequence for lesion quantification (170 sagittal slices, field of view of 240 mm, resolution of 1 × 1 × 1 mm^3^, TR/TE/inversion time in ms of 4800/286/1650, turbo factor of 182, acquisition time of 6min 30s). A structural isotropic T1-weighted 3D sequence (T1 fast field echo; 180 sagittal slices; FOV: 240 mm × 240 mm; voxel size: 1 mm × 1 mm × 1 mm; TR, TE, TI/ms: 10/4.6/1000; flip angle: 8°, turbo factor: 164; acquisition time: 6′00″min) was also included.

### Quantitative Analyses

For this study we used quantitative DTI metrics which were assessed based on a recent preceding study of our group [15]. In that study we conducted TBSS analyses including the participants described above to investigate differences in WM tract integrity between patients and HC. Therein, the detected FA and MD alterations in patients with CIS or RRMS compared to HC were widespread all over the brain and involved most supratentorial and infratentorial WM tracts. FA reductions also involved the cerebellum. AD was increased in patients compared to HC in the right superior corona radiata, while there were no significant RD differences between patients and HC.

**Fig. 1:**
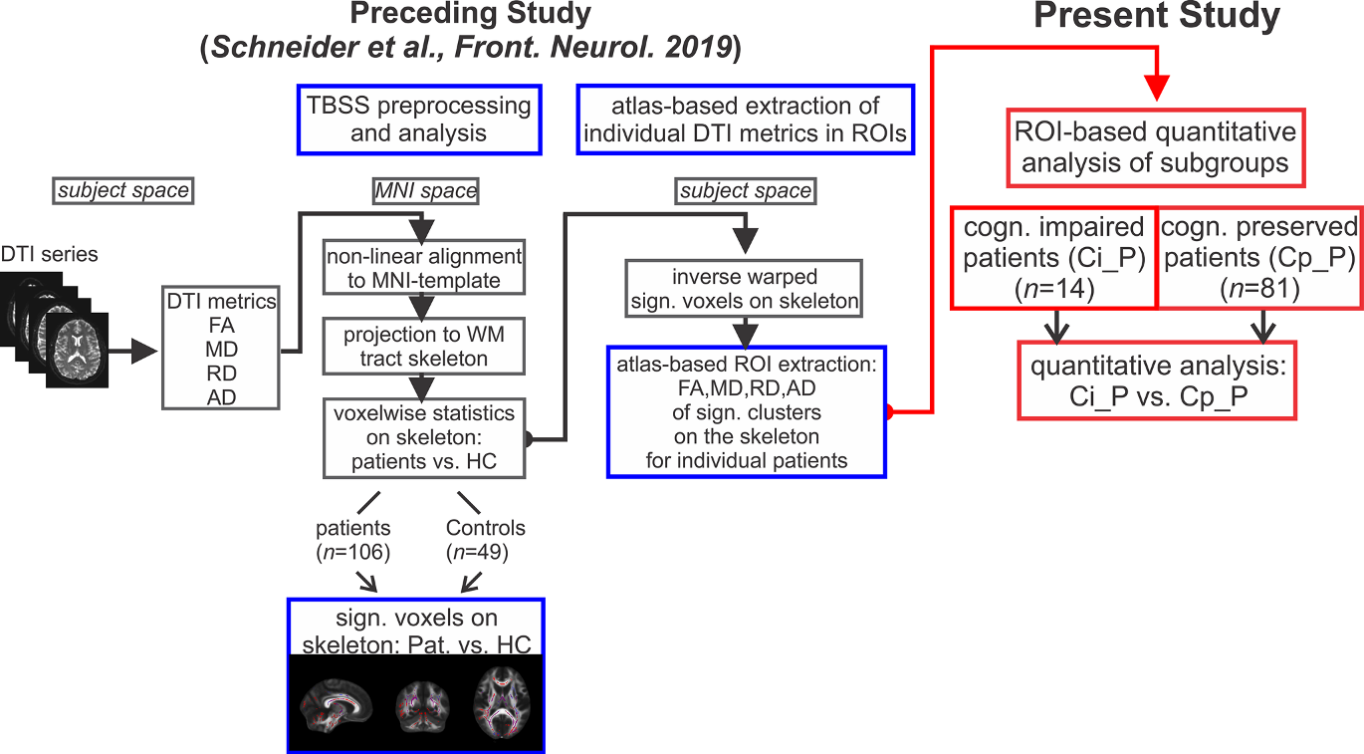
Study design: built on atlas-based region of interest (ROI) extraction the present study investigated (*red* framed elements) quantitative diffusion tensor imaging (DTI) metrics of cognitively impaired patients (Ci_P, *n* = 14) in comparison to cognitively preserved patients (Cp_P, *n* = 81). It was based on the results of a preceding tract-based spatial statistics (TBSS) analysis (*blue* framed elements) which yielded individual fractional anisotropy (FA), mean diffusivity (MD), radial diffusivity (RD) and axial diffusivity (AD) values in clusters on the white matter (WM) skeleton in which significant differences between patients and healthy controls (HC) have been detected [15]. *MNI* Montreal Neurological Institute, *cogn.* cognitive, *Pat*. patients, *sign.* significant, *vs.* versus (compared against)

We extracted individual quantitative results of the DTI metrics for each participant in all clusters in which significant alterations between patients and HC had been found. A summarizing figure showing the significant clusters of FA and MD alterations on the WM tract skeleton is provided as an online resource (Online_Resource_1). In the present study we analyzed these quantitative DTI results to compare Ci_P and Cp_P. Fig. 1 illustrates the entire study design incorporating the preceding analyses and the present study. In the following Sect. 2.4.1 and 2.4.2 we summarize the methodology which was used in the already published preceding study [15]. For further details of the TBSS analysis and atlas-based extraction of DTI metrics in significant clusters on the WM tract skeleton we refer to [15].

#### TBSS Analysis of Patients and Healthy Controls (Summary of [15])

In brief, we used tract-based spatial statistics (TBSS, version 1.2) as a part of the FSL software package [22, 23] with default settings as described in the TBSS manual to perform voxel-wise statistics of DTI data (https://fsl.fmrib.ox.ac.uk/fsl/fslwiki/TBSS/UserGuide; Fig. 1). First, FA, MD, RD and AD images were generated from the raw diffusion-weighted data. Second, FA images were warped to a 1 × 1 × 1 mm^3^ standard space target image (FMRIB58_FA) using nonlinear registration and a mean FA skeleton, which represented the centers of all tracts common to the group was generated. The aligned FA, MD, RD and AD data were projected onto this skeleton and the resulting skeletonized data were used to perform voxel-wise statistics between patients and HC.

Between-group analyses were conducted using permutation methods for non-parametric voxel-wise statistical analysis (FSL randomize tool) [24] (two-sample unpaired t‑tests with age and sex as nuisance variables). We identified regions of the WM tracts showing significant group differences in the TBSS analyses (clusters) with significantly altered DTI parameters using threshold-free cluster enhancement at a significance threshold of *p* < 0.01.

#### Region of Interest (ROI) Based Extraction of Quantitative DTI Metrics (Summary of [15])

For each participant we extracted mean values and standard deviations of the DTI metrics from the relevant clusters using the image statistics utilities in FSL (FSLUTILS: fslmaths, fslmeants, fslstats) by attributing them to regions of the DTI-based brain white matter label atlas (JHU-ICBM-labels-1 mm) [25] and the probabilistic cerebellar atlas (Cerebellum-MNI_fnirt_1 mm, at a 25% threshold) [26]. As detailed in the online resource, the relevant clusters affected all regions of the JHU-label atlas and the main ROIs of the cerebellar atlas which were included in the further analyses.

#### Cognition-related Group Analysis

The resulting quantitative DTI results were further analyzed for the subset of patients who received cognitive testing to assess differences between Ci_P and Cp_P.

#### Lesion Quantification

We generated lesion probability maps for Ci_P and Cp_P subgroups to assess whether there was an overlap between lesions and relevant ROIs extracted from the TBSS analysis. Individual lesion probability maps and total FLAIR lesion volume were obtained for each patient using the lesion prediction algorithm in LST toolbox version 3.0.0 (www.statistical-modelling.de/lst.html) for SPM [27]. During the lesion segmentation procedure, FLAIR images and resulting lesion maps were coregistered to the corresponding 3D-T1-weighted series. The individual T1-weighted series were registered and normalized to the DARTEL template in MNI space using the preprocessing procedures of the CAT12 segmentation tools (CAT12, version R1165, http://www.neuro.uni-jena.de/hbm2016/GaserHBM2016.pdf). Deformation fields of the T1-transformations were applied to the individual lesion probability maps to transform them to the common MNI-space using the SPM12 Normalize (write) function. We generated single, averaged lesion probability maps for both subgroups (Ci_P and Cp_P) using the SPM12 image calculator tool. For visual representation in the FSL image viewer FSLeyes, the lesion probability maps were thresholded at a level of 0.15, showing regions where at least 15% of the patients had FLAIR lesions.

### Statistics

The IBM SPSS software (version SPSS 24) was used for further statistical analyses of quantitative results. For all statistical analyses in SPSS, linear parametric or non-parametric methods were used, and testing was two-tailed with an α‑level of *p* < 0.05. Comparisons of demographic data were assessed by one-way ANOVA (controlled for age and disease duration), Mann-Whitney U‑tests (for EDSS and lesion load) or χ^2^-tests (for numbers of participants). The association between quantitative DTI parameters in the ROIs and performance in cognition was investigated using a general linear multivariate model including CI status and sex as fixed factors, and age and EDSS as covariates. We corrected the resulting *p*-values with *p* < 0.05 for multiple comparisons by applying a Benjamini-Hochberg correction for false discovery rates (FDR at level q = 0.05).

## Results

### Demographic and Clinical Data

Of the patients 14.7% (14/95) were classified as cognitively impaired. The demographic results and characteristics are summarized in Table 1. In accordance with the inclusion criteria, patients had short disease duration, low grades of brain lesion loads and clinical disability (EDSS). In Ci_P the EDSS and brain lesion load were higher than in Cp_P (both: *p* < 0.05). Furthermore, the Ci_P group contained a higher proportion of male patients and patients at higher age than the Cp_P group. The majority of patients in the Ci_P group showed signs of moderate cognitive impairment, as indicated by the median of the MUSIC cognition score of 13 in this group.

**Table 1:** Summary of demographic and clinical data; classification into cognitively impaired (Ci_P) or cognitively preserved (Cp_P) according to screening test (MUSIC cognition) for CI. The columns “All patients” and “healthy controls” refer to the participants of the preceding TBSS analysis [15]

	Ci_P	Cp_P	All patients	HC	*p*-value
***n***	14	81	106	49	–
**Female/male**	6/8	55/26	68/38	32/17	n.s.^a^
**Age** (years) mean ± SD	40 ± 10	36 ± 11	37 ± 11	42 ± 14	Ci_P vs. Cp_P: n. s. All patients vs. HC: *p* < 0.05 ^b^
**Disease duration** (months) mean ± SD	10.6 ± 8.5	12.6 ± 10.4	11.6 ± 10.5	–	n. s. ^b^
**Lesion load** (ml) median (IQR)	2.8 (1.4–5.3)	0.9 (0.2–2.6)	2.1 (0.9–5.3)	–	Ci_P vs. Cp_P : *p* < 0.05 ^c^
**EDSS** median (IQR)	3.0 (1.5–3.5)	1.5 (1.5–2.5)	1.5 (1.5–2.5)	–	Ci_P vs. Cp_P: *p* < 0.05 ^c^
**MUSIC cognition score** median (IQR)	13 (12–18)	26 (23–28)	–	–	Ci_P vs. Cp_P : *p* < 0.001 ^c^

*Ci_P* cognitively impaired patients, *Cp_P* cognitively preserved patients, *SD* standard deviation, *IQR* interquartile range, *n.* *s.* not significant

^a^ χ^2^-square test

^b^ univariate ANOVA

^c^ Mann-Whitney U‑test between Ci_P and Cp_P

### Diffusion Metrics in Cognitively Impaired Patients Compared to Non-impaired Patients

Based on TBSS analysis between all patients and HC and atlas-based extraction of quantitative DTI results, we examined associations with CI for FA, MD and AD in the significant clusters. The analysis included age, sex and EDSS as nuisance variables, since associations between EDSS and FA in different brain structures are well known in MS [14, 28, 29] and there were between-group differences regarding age and the male/female proportion in our cohort.

The FA showed multiple differences between Ci_P and Cp_P. Details are presented in Table 2 which shows FA for Ci_P and Cp_P in those ROIs where differences between the two groups were significant. Therein, FA was decreased in Ci_P compared to Cp_P in supratentorial, midbrain and pontine and cerebellar WM regions. In detail, we found marked FA reductions (*p* ≤ 0.002) in the splenium of CC, the left cerebral peduncle, the cerebellar lobule (right V) and in the right parahippocampal cingulum (cingulum/hippocampus). In addition, FA was reduced in the right fornix (cres)/stria terminalis, left posterior corona radiata and thalamic radiation (including optic radiation), left tapetum, bihemispheric medial lemniscus and cerebellar lobules left I–IV, left V and left VI. The associations with cerebellar lobule left I–IV and left thalamic radiation (incl. optic radiation) did not survive the FDR correction for multiple comparisons. The FA differences in the cerebellar lobule left I-IV and left thalamic radiation (incl. optic radiation) were not significant after FDR correction for multiple comparisons.

**Table 2:** Fractional anisotropy (FA) in patients with cognitive impairment (Ci_P) compared to cognitively preserved patients (Cp_P) patients

FA (mean ± SD)	Cp_P *n* = 81	Ci_P *n* = 14	*p*-value
Splenium of corpus callosum	0.949 ± 0.02	0.918 ± 0.05	0.001*
Tapetum L	0.870 ± 0.08	0.794 ± 0.10	0.019*
Cingulum (hippocampus) R	0.722 ± 0.08	0.657 ± 0.13	0.002*
Fornix (cres)/Stria terminalis R	0.813 ± 0.05	0.776 ± 0.08	0.042*
Posterior corona radiata L	0.731 ± 0.04	0.694 ± 0.05	0.012*
*Posterior thalamic radiation incl. optic radiation L*	*0.832* *±* *0.04*	*0.799* *±* *0.04*	*0.050*
Cerebral peduncle R	0.936 ± 0.02	0.919 ± 0.04	0.012*
Cerebral peduncle L	0.913 ± 0.02	0.888 ± 0.03	< 0.001*
Medial lemniscus R	0.878 ± 0.03	0.858 ± 0.04	0.013*
Medial lemniscus L	0.830 ± 0.04	0.808 ± 0.04	0.024*
*Cerebellum lobules I-IV L*	*0.349* *±* *0.03*	*0.330* *±* *0.02*	*0.095*
Cerebellum lobule V R	0.375 ± 0.02	0.349 ± 0.03	< 0.001*
Cerebellum lobule V L	0.389 ± 0.02	0.368 ± 0.03	0.024*
Cerebellum lobule VI L	0.349 ± 0.03	0.327 ± 0.03	0.009*

Group differences between Ci_P and Cp_P analyzed using a general linear multivariate model controlling for age, sex and EDSS. *: significant after correction for multiple comparisons (Benjamini-Hochberg correction for false discovery rates; FDR at level q = 0.05). *Italics* regions which did not survive FDR correction.

*SD* standard deviation, *FA* fractional anisotropy, *R* right hemisphere, *L* left hemisphere

**Fig. 2:**
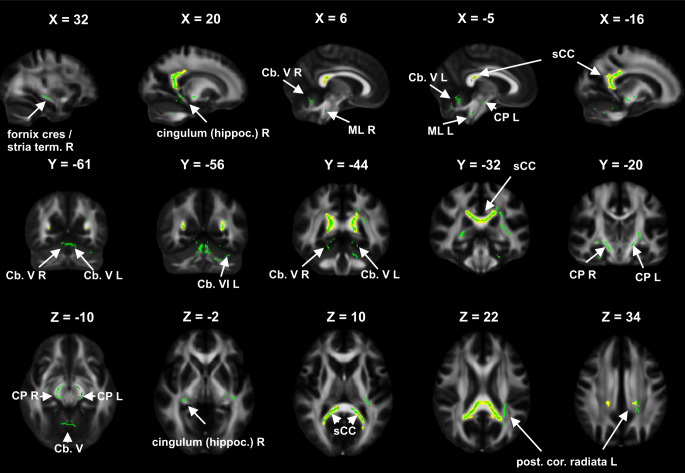
Differences between cognitively impaired patients (Ci_P) and cognitively preserved patients (Cp_P): the figure demonstrates a voxel-wise group comparison, whereby regions of interest with significant fractional anisotropy (FA) reduction (*green *overlay) or mean diffusivity (MD) increase *(yellow *overlay, dilated by 1.5 pixels to improve visibility) in Ci_P compared to Cp_P are shown in sagittal, coronal and axial views on an FA template map in MNI coordinates, X, Y, Z: slices positions in MNI coordinates. The presentation is based on the comparison of the patients and the different groups are not shown here. The *green* and *yellow* overlays highlight those clusters of significant differences between patients and healthy controls (based on the preceding tract based spatial statistics analysis [15]) in which stronger involvement was detected in the Ci_P subgroup. FA reduction and MD increase is visible in the splenium of CC. Additional FA reduction is shown in the left hippocampal cingulum, fornix (cres.)/stria terminalis and posterior corona radiata, bihemispheric cerebral peduncle and medial lemnisculus, and in cerebellar lobules V and VI. *L* left hemisphere, *R* right hemisphere, *sCC* splenium of corpus callosum, *post. cor. radiata* posterior corona radiata, *CP* cerebral peduncle, *Cb* cerebellum, *cingulum (hippoc.)* hippocampal cingulum, *fornix (cres.)/stria term.* fornix (cres.)/stria terminalis, *ML* medial lemniscus

MD was significantly increased in the splenium of CC (*p* = 0.010) in Ci_P (43.6 ± 4.0 10^−5^ mm^2^/s) compared to Cp_P (41.4 ± 2.2 10^−5^ mm^2^/s). Fig. 2 further illustrates the localization of the ROIs in which significantly altered FA or MD have been detected in Ci_P compared to Cp_P. Only those clusters of significantly altered DTI metrics in patients compared to HC (based on the preceding TBSS analysis) have been highlighted in the figure, in which the Ci_P subgroup was more strongly involved than the Cp_P group.

Regarding AD, for which only a single significant cluster had been detected in the TBSS analysis (right superior corona radiata), there were no significant group differences between Ci_P and Cp_P. For RD we had not detected significant clusters in the TBSS analysis.

### Lesion Distribution in Cognitively Impaired Patients and in Non-impaired Patients

**Fig. 3:**
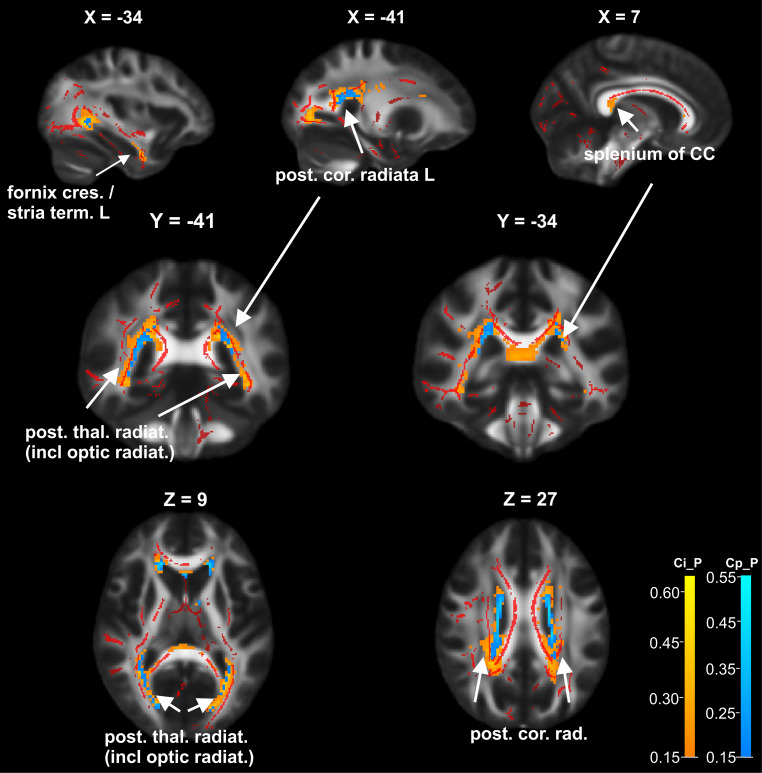
Lesion probability in cognitively impaired patients (Ci_P) and cognitively preserved patients (Cp_P): lesion probability maps of Ci_P (*orange/yellow* overlay) and Cp_P (*light blue* overlay) subgroups, and FA clusters of significant differences between patients and healthy controls (*red* overlay, based on the preceding TBSS analysis [15]) are shown in sagittal, coronal and axial views on an FA template map in MNI coordinates, X, Y, Z: slices positions in MNI coordinates. The lesion probability maps were thresholded at 0.15, thus voxels in which at least 15% of patients had FLAIR lesions are displayed. Areas of high lesion probability which were detected bilaterally in the posterior corona radiata and posterior thalamic radiation were found in both, Ci_P and in Cp_P, with larger involvement in Ci_P. Exclusive lesion load detected in the Ci_P group is depicted in the splenium of the corpus callosum and the bilateral fornix (cres)/stria terminalis, respectively temporal WM. *L* left hemisphere, *R* right hemisphere, *post. cor. rad.* posterior corona radiata, *ant. cor. rad.* anterior corona radiata, *sup. cor. rad.* superior corona radiata, *post. thal. radiat.* *(incl. optic radiat.)* posterior thalamic radiation (including optic radiation), *fornix (cres.)/stria term.* fornix (cres)/stria terminalis

The local distribution of the T2-FLAIR lesions was mainly restricted to periventricular, callosal and temporal regions. Fig. 3 shows the lesion distribution probability maps of Ci_P and Cp_P subgroups and the FA clusters of significant differences between patients and healthy controls (based on the preceding TBSS analysis): lesion probability distributions were similar in both groups with larger involvement in Ci_P (orange) compared to Cp_P (light blue) bilaterally in the posterior corona radiata and posterior thalamic radiation, and exclusive lesion load in Ci_P in the splenium of the corpus callosum (CC) and the bilateral fornix (cres)/stria terminalis or temporal WM. There was only partial overlap between areas of high lesion probability and clusters on the WM skeleton in which the DTI metrics were significantly altered in Ci_P, namely in the splenium of CC, bilateral posterior corona radiata and left hemispheric fornix (cres)/stria terminalis.

## Discussion

In the present monocentric analysis, quantitative DTI results representing microstructural WM alterations showed specific differences between patients with CI and cognitively preserved patients at first diagnosis of MS or CIS. While previous studies using conventional MRI measures could not show an association with CI in early MS [8, 9], our study results demonstrate that quantitative DTI seemed to be more sensitive to detect subtle WM alterations related to CI. Compared with Cp_P, FA was reduced in Ci_P in supratentorial and infratentorial WM regions including cerebellar WM regions. Higher MD in Ci_P compared to Cp_P could be detected only in the splenium of the corpus callosum, while AD did not differ between the two groups. Therefore, we focused the discussion on FA differences. We could identify regional clusters of pronounced FA changes between Ci_P and Cp_P. Therefore, anatomical regions mainly related to cognition with significant changes between the patient groups are discussed separately.

Corpus callosum: our results demonstrated a pronounced involvement of the splenium but not of the body of CC in cognitively impaired patients at disease onset in terms of significantly reduced FA (Table 2) and increased MD (*p* = 0.010). This was analogous to earlier studies on patients with long-standing MS, in which associations between microstructural damage in the CC and CI have been shown [13, 30]. Other studies have reported associations of FA reduction with CI in the splenium and additionally in the body of CC in established MS [14, 28]. Different fiber types that constitute the CC may contribute to a varying vulnerability of callosal regions to microstructural damage: while the splenium is mainly composed of densely packed small diameter fibers, large myelinated fibers are predominantly found in the CC body. Thus, the splenium might be more sensitive to microstructural damage than the body of CC and might be particularly involved in early stages of MS [31]. Previous DTI tractography studies on healthy adults have indicated that information processing speed is probably the main mediator between WM microstructural properties and cognitive performance. Explicitly, FA values of the CC splenium were associated with information processing speed [32, 33]. Accordingly, we hypothesized that in patients with CI at disease onset, WM integrity changes in the splenium of CC are relevant for cognitive tasks which reflect information processing speed.

Parahippocampal cingulum and fornix: furthermore, in Ci_P there was significant FA reduction compared to Cp_P in the right hemispheric parahippocampal part of the cingulum and the fornix (cres)/stria terminals (Fig. 3). Damage of the fornix as the major output of the hippocampus can result in severe memory deficits. Thus, microstructural changes in the fornix were related to reduced verbal memory performance in previous studies. MS patients with better memory performance had higher FA in the fornix than patients with memory deficits [34, 35]. The parahippocampal cingulum contains several hippocampal afferents, and the cingulum as a whole is known to play a critical role in CI because it mediates memory content integration from different parts of the brain [36]. Relations of microstructural changes in the posterior cingulum and fornix reflected by FA reduction and cognitive dysfunction have been shown by Dineen et al. in MS patients with 10-year disease duration [37]. Our results suggest that even in patients at disease onset, CI-related microstructural alterations of fornix and cingulum are detectable. Fornix and cingulum are interrelated structures which are part of the thalamic-hippocampal connections. The interrelation of thalamic atrophy with cognitive performance was shown by Kern et al. who demonstrated that hippocampal-thalamic-prefrontal disruption affected cognitive performance in early MS [38]. These findings were corroborated by a functional connectivity study, which demonstrated reduced connectivity between posterior thalamic nuclei and left supramarginal gyrus, as well as decreased right medial thalamic nuclei connectivity with thalamus and cerebellar areas [39]. In this light, our findings of a pronounced microstructural affection in interrelated thalamic-hippocampal WM regions in cognitively impaired MS patients seem to be conclusive, although we did not investigate thalamic atrophy or brain atrophy in general in the present study.

Cerebellar white matter structures: we additionally detected FA reduction in Ci_P compared to Cp_P in cerebellar structures. Associations between cognition and cerebellar structures have been described in functional MRI studies in the context of corticocerebellar information processing and have also been discussed and corroborated in DTI studies [40, 41]. Especially the cerebellar lobule VI, in which pronounced FA reduction in Ci_P compared to Cp_P was detected in our study, has been suggested to be part of a nonformal cognitive map in the posterior cerebellum contributing to working memory functions and information proceeding speed [40]. Cognitive proceeding speed and working memory capacity, which are equally represented by the MUSIC sum score, are two of the most affected cognitive domains in MS [42]. Our study results confirm the relevant alteration of cerebellar WM microstructures (lobule VI left and lobule V) in early MS and CIS patients with CI.

Cognitive impairment in MS is complex and certainly has multifactorial causes, which cannot be fully explained by altered diffusion metrics. Still, in the present patient group with CIS and early MS, the microstructural alterations in Ci_P were mainly located in tracts which are known to be relevant for cognitive performance, reflecting the neural network character of cognitive functions by involvement of parts of the hippocampal-thalamic-prefrontal network and the corticocerebellar loop. Demyelinating lesions leading to impaired WM tract integrity can be a part of the pathophysiological background of the microstructural observations. In this study, we found only partial overlap between areas of cognitively relevant tract locations and areas of high lesion probability. This indicated that normally appearing WM alterations also contributed to cognitive impairment. Still, the subgroup comparison showed specific areas of high lesion probability in the Ci_P group in the CC splenium and in temporal WM intersected by the fornix (cres)/stria terminalis. Thus, the comparison of Ci_P and Cp_P might be influenced by the differences in the subgroup lesion loads and differences in lesion probabilities between the two groups, as patients with early cognitive impairment might have a higher probability for demyelinating lesions in cognitively relevant structures [43].

We acknowledge limitations of our study. We used a global cognitive performance measure for CI representing the most frequently affected cognitive functions in MS. As a result, we could not assign dysfunctions of more specific cognitive domains to regional WM damage. We rather focused on a characterization of microstructural WM damage of Ci_P compared to Cp_P at the earliest stages of MS. Furthermore, although representing the typical features of a very early MS cohort, the number of cognitively impaired patients was relatively small compared to the cognitively preserved patients. Thus, in future investigations larger numbers of patients, possibly in multicentric analyses, should be included. Furthermore, CI cannot exclusively be attributed to structural impairment of brain WM, due to the known evidence of the associations of cortical lesions and cortical atrophy with CI in RRMS [44], and moreover in progressive MS and patients with longer disease duration [45]. Although contributions of GM pathology to CI have also been reported for CIS and early MS [46], these effects have not been taken into account in this study and must therefore be mentioned as a limitation. A further limitation might be introduced by the definition of the ROIs in which the DTI metrics have been extracted: since the analyses were based on clusters of significant differences between patients and healthy controls gained from the preceding study, and the entire patient group was dominated by Cp_P, regions with specific involvement in Ci_P might have been underestimated or missed by the chosen methodology. Still, the number of those potentially missing regions is probably small since our analyses included regions of nearly all tracts of the JHU-ICBM WM label atlas and most large tracts of the cerebellar atlas. Future studies, including larger numbers of patients should directly investigate the voxel-wise comparison between Ci_P and Cp_P. In addition, we used a 32 gradient direction DTI sequence with limited spatial resolution of 2.5 mm, therefore higher resolution and number of gradient directions would be desirably in future studies. Moreover, future studies should investigate these effects using advanced multi-shell diffusion protocols (e.g. NODDI [47]), since these models measure complementary WM properties and might be more closely related to cognitive performance than DTI [48, 49].

## Conclusion

Referring to our hypotheses, we were able to confirm that microstructural WM alterations were more pronounced in known cognitively relevant tracts of cognitively impaired patients, even in the earliest stages of MS disease. Particularly, the splenium of corpus callosum, fornix, parahippocampal cingulum and cerebellar WM tracts seemed to be relevant for CI. Considering the impact of cognitive impairment on occupational disability and restrictions in quality of life for patients with MS these pronounced alterations of WM integrity in Ci_P at disease onset underline the relevance of early treatment initiation.

## Supplementary Information

Supporting information from the preceding study: group comparison between patients who did or did not undergo cognitive screening in the preceding study, a figure showing the significant clusters of FA and MD alterations on the WM tract skeleton in patients compared to healthy controls
