# Supplementary material for: Microstructural White Matter Alterations in Cognitively Impaired Patients at Early Stages of Multiple Sclerosis
Source: Clin Neuroradiol. 2021 Mar 31;31(4):993–1003. doi: 10.1007/s00062-021-01010-8 (PMC8648694; doi:10.1007/s00062-021-01010-8)
Supplement: Supplementary file 1 — Supporting information from the preceding study: group comparison between patients who did or did not undergo cognitive screening in the preceding study, a figure showing the significant clusters of FA and MD alterations on the WM tract skeleton in patients compared to healthy controls [file 62_2021_1010_MOESM1_ESM.docx]

**Online Resource 1**

**Microstructural white matter alterations in cognitively impaired patients at early stages of Multiple Sclerosis**

Ruth Schneider^1^*, Britta Matusche^2^, Erhan Genç^3^, Ralf Gold^1^, Barbara Bellenberg^2^, Carsten Lukas^2,4^

^1^Department of Neurology, St. Josef Hospital, Ruhr-University Bochum, Bochum, Germany

^2^Institute of Neuroradiology, St. Josef Hospital, Ruhr-University Bochum, Bochum, Germany

^3^Department of Biopsychology, Institute of Cognitive Neuroscience Ruhr-University Bochum, Bochum, Germany

^4^Department of Diagnostic and Interventional Radiology and Nuclear Medicine, St. Josef Hospital, Ruhr-University Bochum, Bochum, Germany

*Correspondence:

Corresponding Author

[Ruth.Schneider@rub.de](mailto:Ruth.Schneider@rub.de)

OCRID 0000-0002-0997-2725

1. **Comparison of patient groups with or without cognitive testing**

In the present study patients who received screening for cognitive impairment (n=95) were selected from a larger patient population (n=106) , for which quantitative DTI metrics had been investigated in a preceding TBSS analysis [Schneider R. et al , 2019]. To confirm that there was no significant selection induced bias in the group of patients who were screened for cognitive impairment, we investigated group differences between the patient groups with or without cognitive testing. The results are shown in table S-1. There were no significant differences regarding age, EDSS or brain lesion load between the groups of patients who did or did not receive cognitive screening.

**Table S-1** Comparison of demographic and clinical data of the patient group who were screened for CI (MUSIC-cognition test) compared to the patients who did not receive cognitive testing. The entire patient population refers to the participants of the preceding TBSS analysis [Schneider R. et al , 2019].

|  | **Patients with cognitive testing** | **Patients without cognitive testing** | **p-value** |
| --- | --- | --- | --- |
| ***n*** | 95 | 11 | - |
| **female/male** | 61/34 | 7/4 | n.s.^a^ |
| **Age** [years]  (mean±SD) | 36±11 | 36±13 | n.s.^b^ |
| **Disease duration** [months] mean±SD | 12.3 ± 10.2 | 6.0 ±12.5 | n.s.^b^ |
| **Lesion load** [ml] median (IQR) | 1.2 (0.3-3.5) | 1.2 (0.8-2.3) | n.s.^c^ |
| **EDSS**  median (IQR) | 1.5 (1.5-3.0) | 1.5 (1.5-2.0) | n.s.^c^ |

^a^ Chi-square test, ^b^ univariate ANOVA, ^c^ Mann–Whitney U-test between patients with or without cognitive testing;

Abbreviations: SD standard deviation, IQR interquartile range, n.s. not significant (p > 0.050)

1. **Results of the preceding TBSS analysis [Schneider R. et al , 2019]: Clusters of significant FA and MD alterations in 106 patients compared to 49 healthy controls**


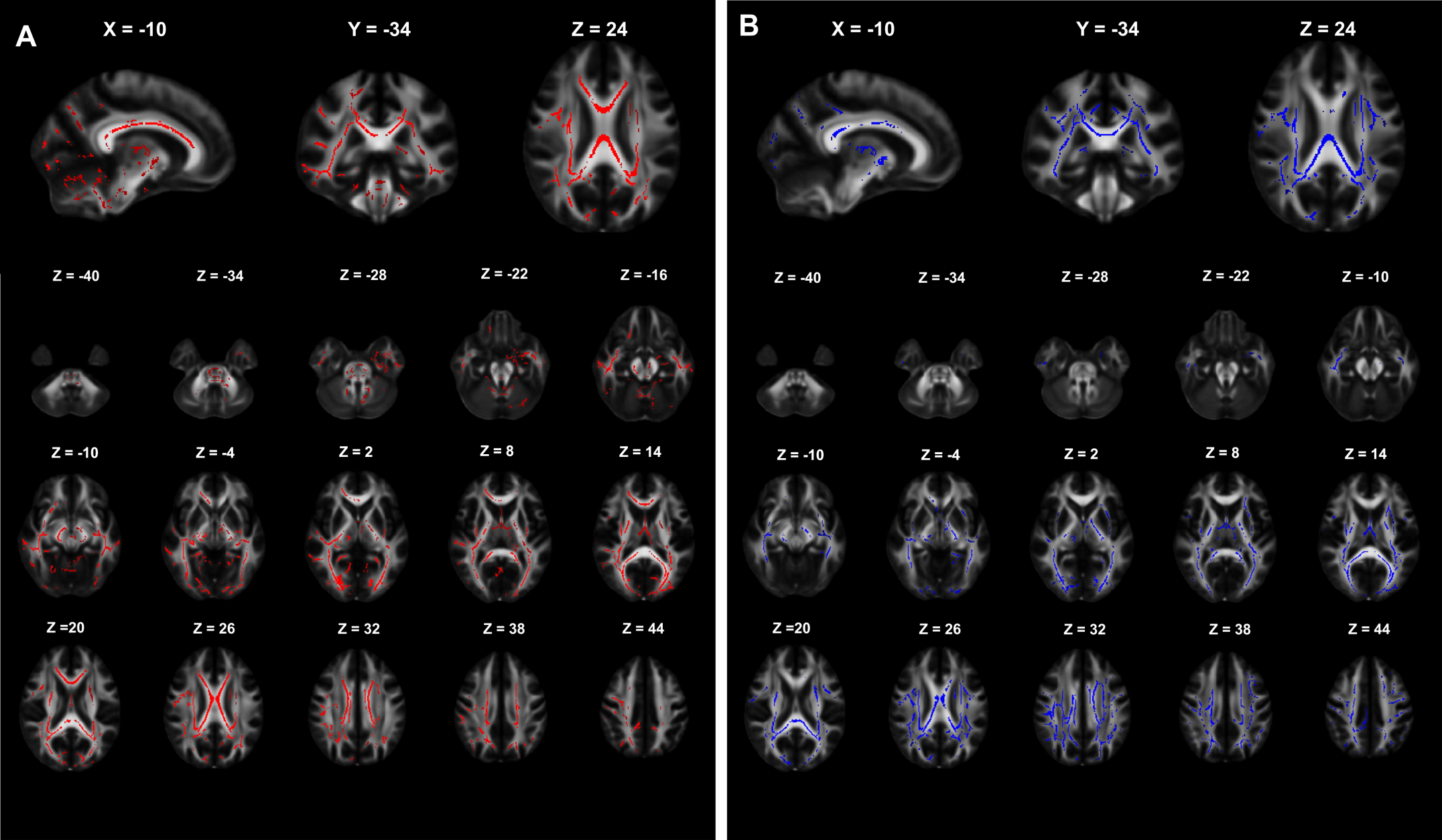
**Fig. S-1** Clusters of significant FA reductions (A) and MD increase (B) in 106 patients (clinically isolated syndrome or MS at disease onset) compared to 49 healthy controls involving most supratentorial WM tracts for FA and MD. Infratentorial FA reduction is visible in brainstem and cerebellar WM tracts. Details of the TBSS analysis and results are available in *Schneider, R., et al., Temporal Dynamics of Diffusion Metrics in Early Multiple Sclerosis and Clinically Isolated Syndrome: A 2-Year Follow-Up Tract-Based Spatial Statistics Study. Front Neurol, 2019. 10: p. 1165.* *doi: 10.3389/fneur.2019.01165.*

1. **Reference**

Schneider R, Genç E, Ahlborn C, Gold R, Lukas C, Bellenberg B. Temporal Dynamics of Diffusion Metrics in Early Multiple Sclerosis and Clinically Isolated Syndrome: A 2-Year Follow-Up Tract-Based Spatial Statistics Study. Front Neurol. 2019;10:1165. doi:10.3389/fneur.2019.01165.
